# Transcriptome Profiling Analysis of Bovine Vaginal Epithelial Cell Response to an Isolated *Lactobacillus* Strain

**DOI:** 10.1128/mSystems.00268-19

**Published:** 2019-09-10

**Authors:** Chunyu Niu, Chao Cheng, Yanmin Liu, Shaolei Huang, Yanru Fu, Peifeng Li

**Affiliations:** a Key Laboratory of Clinical Diagnosis and Treatment Techniques for Animal Disease, College of Veterinary Medicine, Inner Mongolia Agricultural University, Hohhot, China; b Inner Mongolia ShuangQi Pharmaceutical Co., Ltd., Hohhot, China; c Hohhot Vocational College, Hohhot, China; University of Chicago

**Keywords:** primary vaginal epithelial cell culture, *Lactobacillus*, transcriptome profiling, gene expression, RT-qPCR

## Abstract

*Lactobacillus* strain SQ0048 isolated from bovine vagina has been shown to exhibit specific adherence to the epithelium and to produce inhibitory substances; however, the underlying mechanisms remain unclear. We cultured and identified primary bovine vaginal epithelial cells treated with SQ0048 to investigate the pathways involved in host cell responses using transcriptome sequencing (RNA-seq). Transcription profiling showed 296 significantly altered differentially expressed genes (DEGs), of which 170 were upregulated and 126 downregulated. Gene Ontology (GO) enrichment analysis of the DEGs revealed significant enrichment of 424 GO terms throughout the differentiation process (*P < *0.05). Kyoto Encyclopedia of Genes and Genomes (KEGG) analysis showed that the DEGs were successfully annotated as members of 171 pathways, with 23 significantly enriched KEGG pathways (*P < *0.05). A relatively high number of genes were enriched for the endoplasmic reticulum protein processing and interleukin-17 (IL-17) signaling pathways and for antigen processing and presentation. DEGs were verified by quantitative reverse transcription-PCR (RT-qPCR) and determination of which were most enriched for endoplasmic reticulum protein processing pathways, the activation of which might be a major factor underlying *Lactobacillus* adhesion to cells and pathogenic inhibition.

**IMPORTANCE** Bovine bacterial vaginitis causes infertility, abortion, and postpartum uterine diseases, causing serious economic loss to the dairy industry. The large-scale use of antibiotics destroys normal genital tract flora and hinders the defense mechanisms of the host. Recent research suggests that lactobacilli present in the vaginal microflora of healthy cows constitute the primary microbiological barrier to infection by genital pathogens, exerting a protective role on the reproductive tract via specific adherence to the epithelium and the production of inhibitory substances. Our research identified the mechanisms for *Lactobacillus* adhesion and pathogenic inhibition, providing valuable information for the development of new probiotics and the discovery of novel therapeutic targets for the prevention of infections in dairy cows.

## Abstract (precis)

Bovine bacterial vaginitis causes infertility, abortion, and postpartum uterine diseases, causing serious economic loss to the dairy industry. The large-scale use of antibiotics destroys normal genital tract flora and hinders the defense mechanisms of the host. Recent research suggests that lactobacilli present in the vaginal microflora of healthy cows constitute the primary microbiological barrier to infection by genital pathogens, exerting a protective role on the reproductive tract via specific adherence to the epithelium and the production of inhibitory substances. Our research identified the mechanisms for *Lactobacillus* adhesion and pathogenic inhibition, providing valuable information for the development of new probiotics and the discovery of novel therapeutic targets for the prevention of infections in dairy cows.

## INTRODUCTION

Microbes that commonly infect the reproductive tract of cattle are known to cause infertility, abortion, and postpartum uterine diseases, causing serious economic loss to the dairy industry (1). At present, antibiotics are widely used in the treatment of reproductive tract infections in dairy cows. The large-scale use of antibiotics to combat these infections results in economic and health disadvantages such as increased production costs, drug residues in animal food, development of microbial resistance to antibacterial drugs, and destruction of normal genital tract flora, causing failure in the defense mechanisms of the host (2, 3). Therefore, effective and nonresidual alternative therapies for the prevention and treatment of bovine bacterial vaginitis are urgently needed for the maintenance and development of a robust dairy cow industry.

Lactobacilli present in the vaginal microflora of healthy cows are considered the primary microbiological barrier to infection by genital pathogens (4). In both humans and animals, lactobacilli are the most prevalent and often numerically dominant microorganisms, offering substantial probiotic function and beneficial effects on vaginal health (5, 6). Lactobacilli exert a protective role on the reproductive tract primarily through a combination of specific adherence to the epithelium and the production of inhibitory substances (7, 8). As the predominant microorganisms of the vaginal microbiota, lactobacilli prevent pathogen colonization and enhance host immunity by the production of antagonistic substances such as lactic acid, H_2_O_2_, and bacteriocins (9, 10). Therefore, the dominance of lactobacilli in the microbiota appears to be a good biomarker for a healthy vaginal ecosystem. One study showed that the isolation of bovine vaginal *Lactobacillus* strains revealed differential surface properties and that these strains can be used as probiotics in the prevention and treatment of bovine reproductive tract disease (11, 12). In addition, it has been demonstrated that *Lactobacillus* improves vaginal health in animals and promotes their productivity (13). The specific adherence of *Lactobacillus* to the epithelium contributes to its colonization in the host, enhancing the signal exchange between *Lactobacillus* and host cells, inhibiting the colonization of pathogens, and improving overall immunity (14, 15). Hence, elucidation of the mechanisms involved in the interactions between *Lactobacillus* and host cells is crucial to understanding how *Lactobacillus* inhibits pathogenesis.

Transcription profiling can be used to assess the effect of transcriptional changes to epithelial cells upon treatment with *Lactobacillus* at the whole-genome scale. Studies on transcription profiling in host cells treated with *Lactobacillus* have been conducted previously. Taranu et al. reported the protective role of lactobacilli in epithelial barrier function against inflammation and in the activation of immune response (16). A large number of genes related to the immune system were obtained by transcriptome profiling. Jacouton et al. studied the influence of Lactobacillus rhamnosus in intestinal epithelial cells with transcriptome profiling (17), revealing that Lactobacillus rhamnosus induced differential gene expression in human small intestinal epithelial cell (IECs) and that this effect was accompanied by transcriptome modulation of several pathways, including immune response and metabolism *in vitro*. Consequently, expression profile analysis of genes in bovine vaginal epithelial cells (BVECs) treated with *Lactobacillus* may help explain the mechanisms of specific adherence and identify immune response signaling pathways.

Based on the natural benefits of indigenous microbiota, we previously isolated and identified a dominant *Lactobacillus* strain known as SQ0048 from the vaginal tracts of cattle that has a homology of 99% with Lactobacillus johnsonii; its probiotic potential was also studied (18). Moreover, we subsequently demonstrated its high adhesion ability (19). Following these initial studies, we deemed it necessary to investigate the mechanisms of the specific adherence function of the SQ0048 strain as well as its pathogenic inhibition mechanisms, while looking for possible signaling pathways. Thus, in the present study, we performed transcriptome profiling to determine possible pathogenic inhibition mechanisms in SQ0048 strain-treated epithelial cells. We further analyzed the transcripts by matching them against the Gene Ontology (GO) databases and the Kyoto Encyclopedia of Genes and Genomes (KEGG). The differentially expressed genes (DEGs) and different signaling pathways obtained from the transcription data will provide a test basis for the research and development of probiotic products, while informing new ways to prevent infectious diseases through the restoration of indigenous microflora.

## RESULTS

### Culture characteristics of bovine vaginal epithelial cells.

**FIG 1:**
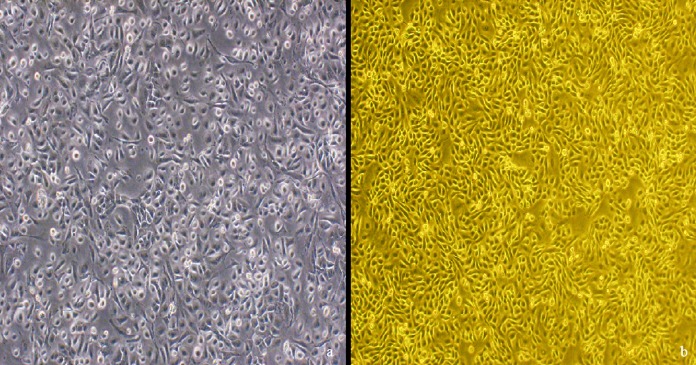
Morphology of bovine vaginal epithelial cells. (a) The primary epithelial cells on day 7 mixed with a small quantity of fibroblasts (200×). (b) The subcultured cells at passages 6 present classic cobblestone morphology (200×).

Cell-cell adherence occurred after 48 h, and the BVECs were easily distinguished by rounded or polygonal morphology. After 3 days in culture, the cell proliferative capacity was significantly improved and the cells began to gather in groups. The epithelial cell confluence reached 70 to 80% on day 7 (Fig. 1a). The subcultured cells began to adhere after 12 h and reached 70 to 80% confluence on day 3, showing a higher growth rate than that of primary cells. Furthermore, at passages 6 and 7, cell proliferative capacity was stable and cell-cell contact was evident, presenting classic cobblestone morphology (Fig. 1b).

### Immunofluorescence analysis.

**FIG 2:**
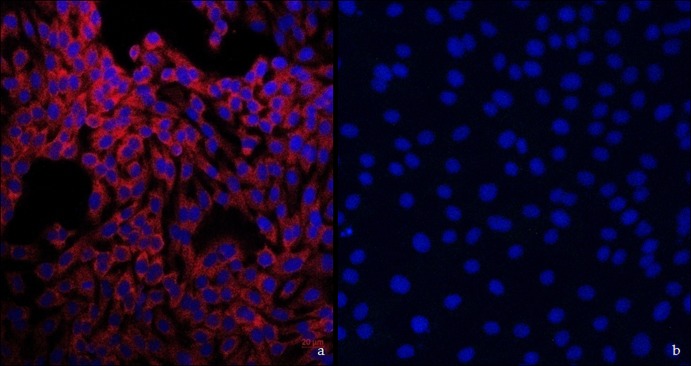
Immunocytochemistry of BVECs. (a) BVECs expressing cytokeratin-18 (red). (b) BVECs negative-control group; nuclei with DAPI staining (blue).

Immunofluorescence staining showed cobblestone-like cells expressing cytokeratin-18, indicating that they were of epithelial origin. We isolated BVECs that showed strong red fluorescence staining in cytoplasm, corresponding to cytokeratin-18 expression (Fig. 2a). Cytokeratin-18 expression was not observed in the BVECs of the negative-control group (Fig. 2b).

### Classification of transcriptional sequencing data.

To evaluate the transcriptional response of the BVECs to treatment with the SQ0048 strain and seek out which host factors were involved in the adhesion and inflammatory immune response, we utilized the Illumina HiSeq 4000 platform with cDNA libraries of BVECs treated with SQ0048. As shown in Table 1, a total of 50,431,262 raw reads with a *Q*_20_ value of 99.68% were generated for the treated BVECs and 54,202,017 raw reads with a *Q*_20_ value of 99.64% were generated for the control cells; *Q*_20_ is the number of bases with a phred score of >20 in the raw data as a percentage of the total number of bases. After removing the short reads, low-quality sequence and ambiguous nucleotides were removed from the raw reads, leaving 49,813,706 and 53,421,825 valid reads, respectively, from treated and control BVEC groups that were used for further analysis. All valid reads were aligned to the bovine genome using Hisat 2.0. Furthermore, 47,650,558 and 51,209,330 uniquely mapped reads were obtained from groups of treated and control BVECs, respectively. The uniquely mapped read ratios of 77.28 and 78.08 were determined after filtering adapters and trimming ambiguous results. The results suggested that the high-quality sequencing data could be used for further analysis.

**TABLE 1:** Statistical summary analysis of RNA-seq data sets of treated cells and control cells

Sample	Means for raw reads			Means for mapping		
	No. of raw reads	No. of valid reads	*Q*_20_ (%)	No. of map reads	No. of unique mapped reads	Uniquely mapped ratio (%)
Control cells	54,202,017	53,421,825	99.64	51,209,330	41,710,001	78.08
Treated cells	50,431,262	49,813,706	99.68	47,650,558	38,488,506	77.28

### Analysis of differentially expressed genes.

**FIG 3:**
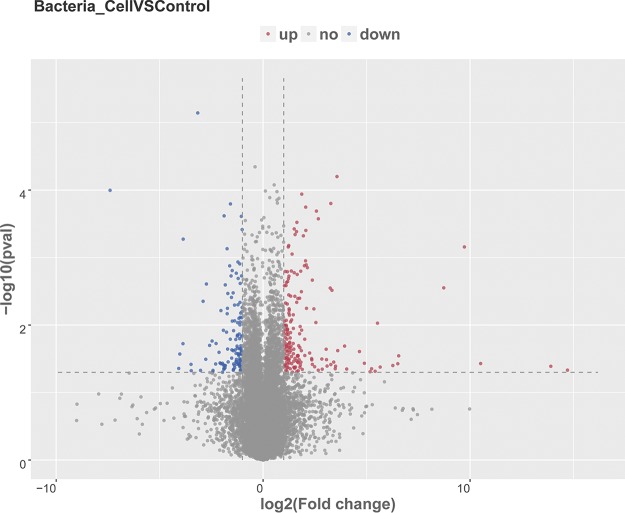
Volcano graph of 296 differentially expressed genes between two groups. Red represents upregulated DEGs; blue represents downregulated DEGs.

BVECs treated with the SQ0048 strain showed a greater degree of differential expression. Differential analysis of the transcript expression profiles demonstrated that 296 genes were significantly altered (≥2-fold change, *P < *0.05), of which 170 were upregulated and 126 downregulated (Fig. 3 shows the volcano graph of 296 differentially expressed genes [DEGs]).

### Functional enrichment analysis of differentially expressed genes.

**FIG 4:**
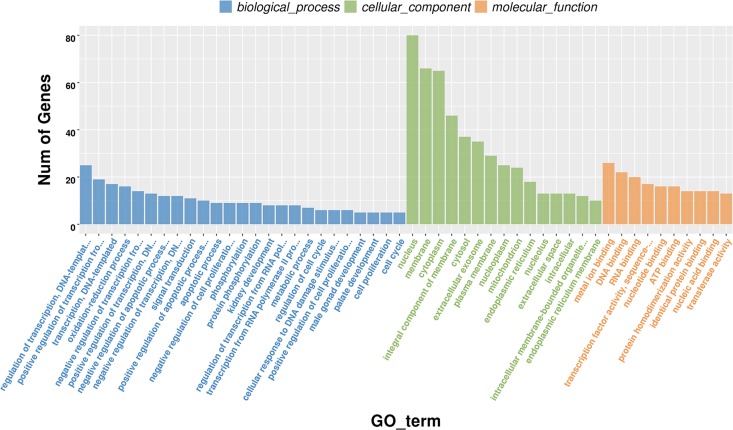
GO classifications of genes in BVECs. The abscissa is the GO classification, and the ordinate is the number of genes in each category.

The transcriptome sequencing (RNA-seq) analysis revealed a total of 18,014 genes, of which 296 genes were significantly differentially expressed. To assess the biological functions of the 296 DEGs, enrichment analysis of GO classification (http://www.geneontology.org/) and KEGG pathway (http://www.genome.jp/kegg) was conducted. GO enrichment analysis of the DEGs revealed a significant enrichment of 424 GO terms throughout the differentiation process (*P < *0.05). The significant GO terms were divided into three major categories—biological process, cellular component, and molecular function—as shown in Fig. 4. Among the biological processes, 178 of the DEGs were distributed to regulation of transcription, oxidation reduction processes, positive regulation of NF-κB transcription factor activity, negative regulation of Jun kinase activity, positive regulation of the p38 mitogen-activated protein kinase (MAPK) cascade, and activation of cysteine-type endopeptidase activity. In the cellular component field, 188 DEGs belonged to the integral component of the peroxisomal membrane, the integral component of the endoplasmic reticulum (ER) membrane, and the signal recognition particle receptor complex. In molecular function, 164 DEGs were responsible for transcription factor activity and cyclic AMP (cAMP)-dependent protein kinase activity.

**FIG 5:**
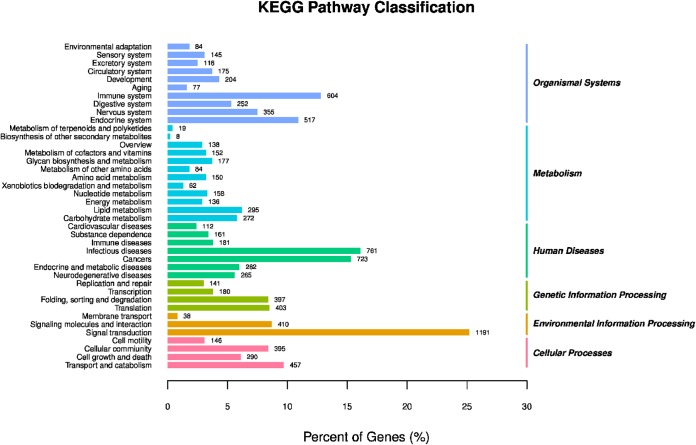
KEGG pathway classification of genes in BVECs. The ordinate is the name of the KEGG metabolic pathway, and the abscissa is the ratio of the number of genes annotated to the pathway and the number of genes in the annotated genes.

**FIG 6:**
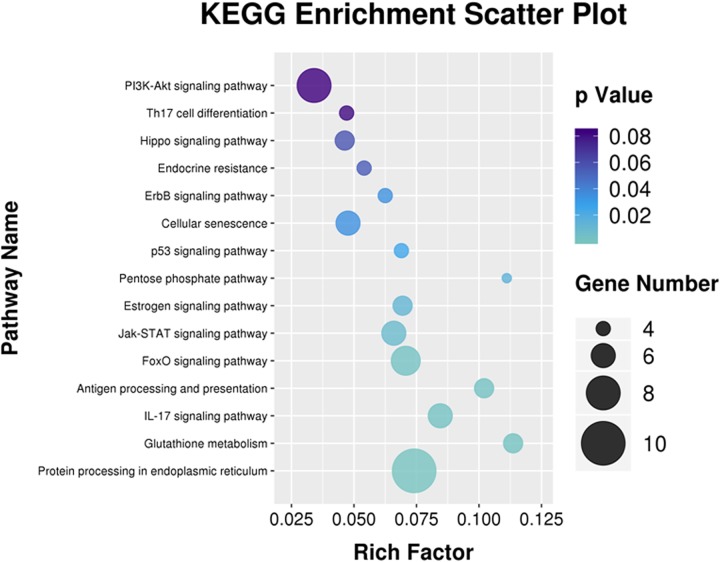
The significantly enriched KEGG pathway of DEGs. Rich factor, number of differentially expressed genes/total number of genes in this KEGG pathway. The larger the value, the greater the enrichment.

The analysis of pathway enrichment allowed for deep recognition of the biological functions of the DEGs. KEGG analysis showed that the DEGs were mainly involved in signal transduction, the immune system, endocrine system, transport, and catabolism, as well as metabolism and genetic information processing (Fig. 5). The DEGs were successfully annotated as members of 171 pathways, and there were 23 significantly enriched KEGG pathways (*P < *0.05). Relatively high numbers of genes were involved in protein processing in the endoplasmic reticulum, phosphatidylinositol 3-kinase (PI3K)-Akt signaling pathway, FOXO signaling pathway, interleukin-17 (IL-17) signaling pathway, antigen processing and presentation, Jak-STAT signaling pathway, p53 signaling pathway, osteoclast differentiation, the ErbB signaling pathway, and other regulatory pathways (Fig. 6).

DEGs were analyzed with KEGG to predict and study the functions of the encoded proteins that were engaged in the primary and most interesting pathways, as well as those that were possibly relevant to *Lactobacillus* adhesion mechanisms and immune response. In pathways associated with the response of the BVECs treated with *Lactobacillus*, approximately 15 DEGs were found (Table 2). Among the DEGs, 10 genes (CKAP4, DNAJB1, DNAJA1, HSPH1, HSPA8, SEC62, PPP1R15A, DERL2, HSP90AA1, and HSPA1A) were upregulated and involved in protein processing in the endoplasmic reticulum—the pathway in which most of the DEGs participated. Five DEGs (FOSL1, JUN, IL17RA, FOSB, and HSP90AA1) were upregulated and belonged to the IL-17 signaling pathway, and four (CD74, HSPA8, HSP90AA1, and HSPA1A) were upregulated and participated in antigen processing and presentation.

**TABLE 2:** Significantly upregulated or downregulated genes involved in signaling in BVECs

Gene name	Gene description	Fold change	*P* value	Regulation
CKAP4	Cytoskeleton-associated protein 4	35.99	0.04913	Up
HSPH1	Heat shock protein family H (Hsp110) member	5.48	0.00575	Up
SEC62	Translocation protein SEC62	2.03	0.01760	Up
PPP1R15A	Protein phosphatase 1 regulatory subunit 15A	3.65	0.00011	Up
DERL2	Derlin 2	2.02	0.03959	Up
HSPA8	Heat shock protein family A (Hsp70) member 8	3.08	0.0046	Up
DNAJB1	DnaJ heat shock protein family (Hsp40) member B1	10.66	0.03533	Up
DNAJA1	DnaJ heat shock protein family (Hsp40) member A1	2.6	0.00838	Up
HSP90AA1	Heat shock protein 90 alpha family class A member 1	2.17	0.01007	Up
HSPA1A	Heat shock 70-kDa protein 1A	847.97	0.00069	Up
FOSL1	FOS-like 1, AP-1 transcription factor subunit	3.61	0.00320	Up
JUN	Jun proto-oncogene	11.89	0.00006	Up
IL17RA	Interleukin-17 receptor A	2.21	0.03215	Up
FOSB	FosB proto-oncogene	2.74	0.01874	Up
CD74	CD74 molecule	2.79	0.03432	Up

### Validation of selected genes by RT-qPCR.

**FIG 7:**
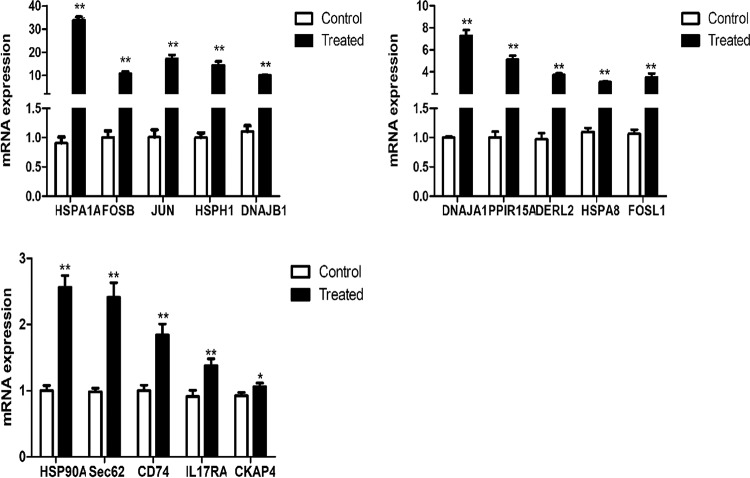
Relative quantification of DEGs for verification by RT-qPCR. RT-qPCR relative expression levels of selected genes were chosen for the cells treated with SQ0048. The expression of each gene was normalized to the average expression of the endogenous reference gene β-actin. *, *P* < 0.05; **, *P *<* *0.01.

To validate the results of RNA-seq analysis, quantitative reverse transcription-PCR (RT-qPCR) was used to examine DEGs with significant roles in adhesion mechanisms and immune response that were upregulated. The expression trends of 15 DEGs (HSP90AA1, HSPA1A, FOSL1, JUN, IL17RA, CKAP4, DNAJB1, DNAJA1, HSPH1, HSPA8, SEC62, PPP1R15A, DERL2, FOSB, and CD74) were found to be similar to those obtained by RNA-seq, suggesting that the RNA-seq data reliably reflected the gene expression trends (Fig. 7).

## DISCUSSION

The vaginal microbiota can be altered by external factors such as environmental exposures, microbial interspecies competition, and commensalism (1), with the microbial species that inhabit the vaginal tract playing an important role in the maintenance of health and prevention of infection. It is recognized that a spectrum of microbial profiles can produce a stable vaginal ecosystem with the ability to prevent vaginitis and other reproductive tract infections (20). *Lactobacillus*, which is dominant among bovine vaginal microbial species, benefits the epithelium with regard to cell proliferation and survival, prevention of epithelial injury, and improvement of the epithelial barrier and immune function (21–23). Adherence of vaginal lactobacilli to host cells has been shown to prevent colonization by pathogenic microorganisms *in vitro* and to stimulate host defense mechanisms against pathogens (12, 24). However, although the health benefits of lactobacilli isolated from bovine vagina are widely recognized, the molecular mechanisms of adhesion and pathogenic inhibition have yet to be determined. Therefore, we investigated the transcriptome profile of differential gene expression in BVECs treated with the SQ0048 strain.

In this study, we cultured primary BVECs confirmed by morphology and immunofluorescence. Meanwhile, using transcriptome profiling analysis, we showed that the BVECs treated with the SQ0048 strain distinctly modulated a high number of genes encoding different processes. We identified 296 DEGs in SQ0048 strain-stimulated BVECs compared to nonstimulated BVECs. To confirm the results of the transcriptome profiling analysis, 15 genes were chosen and their expression was examined by RT-qPCR. DEGs encode proteins that are involved in transcription, signaling, the cell cycle, protein folding, and the inflammatory response. Furthermore, according to the GO classification and KEGG pathway enrichment analysis, we found that the DEGs were mainly enriched in several related pathways, including protein processing in the endoplasmic reticulum, the IL-17 signaling pathway, and antigen processing and presentation.

The IL-17 signaling pathway is critical for host defense and contributes to the pathogenesis of autoimmune diseases and cancer (25). Huang et al. demonstrated that IL-17R signaling is required for host defense against extracellular bacterial and systemic Candida albicans infections (26). IL-17A is required for host inhibitory pathogens (27). Antigen processing and presentation pathways are related to inflammation and defense against infectious agents (28). Wang et al. have reported that in the microbial infection of host cells, exosomes take part in antigen presentation for the activation of immune cells and stimulate the release of inflammatory factors and the expression of immune molecules, thus modulating the immune responses of host cells (29).

**FIG 8:**
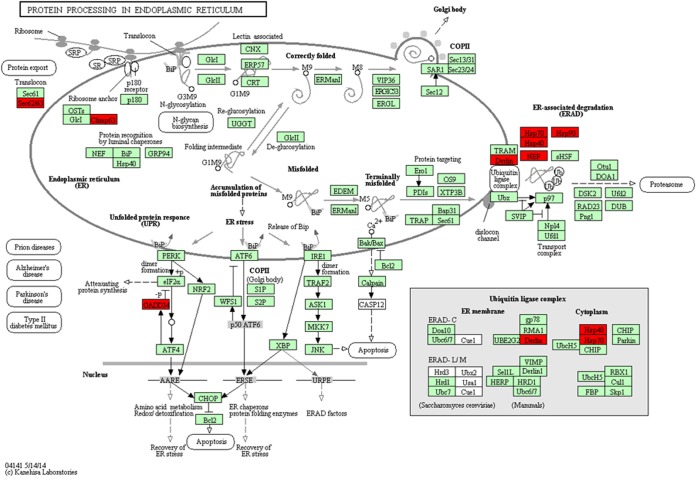
Identification of DEGs relevant to protein processing in the endoplasmic reticulum pathway. Red boxes refer to genes with an associated unigene(s) that was/were upregulated in the BVECs.

Protein processing in the endoplasmic reticulum regulates fundamental cellular functions such as the homeostatic maintenance of stress response and protein folding (30). Interestingly, in our study, protein processing in the endoplasmic reticulum was the most enriched pathway in that most of the DEGs related to the specific adherence function participated in this pathway. The ER serves to maintain protein homeostasis, including the regulation of the concentration, conformation, folding, and trafficking of client protein in cells (31). It plays crucial roles in cellular homeostasis by controlling the processing and folding of secretory and membrane proteins. Under normal physiological conditions, the ER transports only correctly folded proteins to the Golgi apparatus, while misfolded proteins are extracted for ubiquitin-dependent ER-associated degradation (ERAD) (32). Many studies have reported that the viral infection by and replication of *Flaviviridae* such as Japanese encephalitis virus, bovine viral diarrhea virus, and hepatitis C virus induce ER dysfunction and stress (33, 34). Another study demonstrated that Brucella melitensis was a facultative intracellular bacterium that fuses with the ER to replicate, resulting in a marked reorganization of the ER around the replicating bacteria and triggering the unfolded protein response (UPR) (35). As mentioned above, bacteria and viruses cause ER stress. In the present study, the transcriptome profiling results showed that incubation of BVECs with *Lactobacillus* also activates protein processing in the endoplasmic reticulum pathway (Fig. 8). Transcription profiling illustrated that DNAJA1, HSPA1A, HSP90AA1, Sec62, and other key components involved in protein processing in the endoplasmic reticulum pathway were positively regulated. Chaperones are specialized proteins that play a key role in cellular homeostasis by assisting in protein folding, assembly of macromolecular complexes, protein transport, and cellular signaling (36). Heat shock protein 90 (Hsp90) is an essential molecular chaperone that forms a large multichaperone complex that mediates the proper folding, activation, and assembly of its numerous substrate, or “client,” proteins (37). The molecular chaperone Hsp90 orchestrates the regulatory circuitry governing fungal morphogenesis, biofilm development, drug resistance, and virulence. One study demonstrated that Hsp90 was associated with the rapid development of antifungal resistance in the yeast Candida albicans (38). Montanari et al. observed that host cellular Hsp90 expression and activity interfere with adhesive and invasive events driven by NadA (39). HSP70, alias HSPA1A, serves as a molecular chaperone, playing a pivotal role in the protein quality control system, ensuring the correct folding of proteins and the refolding of misfolded proteins and controlling the targeting of proteins for subsequent degradation (40). Ghazaei (41) showed that the adhesion of bacteria to host cells is mediated by both the host and bacterial Hsp70. Following infection of the host, bacterial Hsp70 (DnaK) initiates bacterial survival processes and triggers an immune response by the host. Meanwhile, Hsp70 is bound to both host and microbial cell surface membranes and therefore facilitates the easy attachment and colonization of pathogens (41). Another molecular chaperone, Sec62, is an essential component of the posttranslational translocation machinery in the ER. A study identified Sec62 as a critical molecular component in the maintenance and recovery of ER homeostasis (42). DNAJA1 is an essential cochaperone of mammalian heat shock cognate 70 protein. It is the primary chaperone that interacts with nascent polypeptide chains and functions to prevent their premature release from the ribosome and misfolding before it is targeted by Hsp70 (41, 43). The results of our transcriptome profiling study indicated that related genes that were upregulated participated in protein processing in the endoplasmic reticulum pathway. Thus, we speculate that activation of this signaling pathway might be a major factor causing *Lactobacillus* adhesion to cells and pathogenic inhibition.

Our results emphasize the importance of the novel candidate pathway involving protein processing in the endoplasmic reticulum, the IL-17 signaling pathway, and antigen processing and presentation. The protein processing pathway in the endoplasmic reticulum is related to the specific adherence function of the SQ0048 strain and the inhibition of pathogenic colonization. The IL-17 signaling pathway and antigen processing and presentation are related to anti-inflammation and immunomodulation activity. However, further studies are necessary to confirm these results and to understand the underlying mechanisms of *Lactobacillus* action *in vivo*.

### Conclusions.

Through the application of a transcriptome profiling strategy, we were able to assess and recognize the genes and complicated pathways involved in the adhesion of the SQ0048 strain and the inhibition of pathogenic colonization. Based on the results of our transcriptome analysis, there was a total of 296 DEGs involved in the pathways of protein processing in the ER (CKAP4, DNAJB1, DNAJA1, HSPH1, HSPA8, SEC62, PPP1R15A, DERL2, HSP90AA1, and HSPA1A), the IL-17 signaling pathway (FOSL1, JUN, IL17RA, FOSB, and HSP90AA1), and the antigen processing and presentation pathways (CD74, HSPA8, HSP90AA1, and HSPA1A). These significantly altered pathways suggested that cells treated with the SQ0048 strain were involved in the activation of protein fold repair and inflammation mediation. In conclusion, our study provides new insights into the host cell responses that are involved in the molecular signaling pathway interactions between bacteria and BVECs by transcriptome profiling. It contributes valuable information for the development of new probiotics and the discovery of novel therapeutic targets for the prevention of infections in dairy cows.

## MATERIALS AND METHODS

### Culture of primary bovine vaginal epithelial cells.

Primary BVECs were derived from fresh vaginal tissues of healthy young cows which were certified by a veterinarian for dairy farming. This study was approved by the Animal Welfare and Research Ethics Committee at Inner Mongolia Agriculture University. Epithelial cells from the bovine vaginal tissues were separated using a procedure described previously (44). Bovine vaginal tissue was maintained on ice packs and transported to the laboratory within 1 to 1.5 h of collection. The vaginal tissue was washed several times with precooling Dulbecco’s phosphate-buffered saline (DPBS) (Gibco/Thermo Fisher Scientific, Waltham, MA, USA), cut into 2-cm^3^ sections, and washed with DPBS solution containing penicillin (100 U/ml) and streptomycin (100 U/ml) until clean. Chain protease (0.1%) was used to further disaggregate the fragments at 4°C for 16 h, and then the tissue was gently scraped into 5 ml of DPBS solution and centrifuged at 1,500 rpm for 5 min. Sediment was harvested by centrifugation and washed with DPBS solution three times. Finally, the isolated BVECs were resuspended in DMEM–F-12 medium (Gibco) containing 15% fetal bovine serum (ExCell Biology, Shanghai, China), 100 U/ml penicillin, and 100 mg/ml streptomycin and incubated in 5% CO_2_ at 37°C. The medium was renewed routinely every other day. Cell growth and morphological characteristics were observed with an inverted microscope (Philips, Tokyo, Japan). After reaching 70 to 80% confluence, cells were detached by 1 ml of 0.25% trypsin-EDTA (Gibco) and inoculated at a split ratio of 1:2 for subsequent experiments.

### Immunofluorescence assay.

To verify the epithelial origin and purity of the isolated BVECs, immunocytochemical staining was conducted. Cytokeratin-18 is known to be an important cytoskeletal component of epithelial cells (45, 46). Cells at logarithmic growth phase were seeded at a density of 1 × 10^5^ cells/ml into 35-mm glass-bottom dishes (Shengyou Biotechnology, Hangzhou, China). After reaching 70 to 80% confluence, the cells were fixed for 20 min with 4% paraformaldehyde (Solarbio, Beijing, China) at 20°C and washed three times with PBS. After fixation, the cells were incubated with 0.25% Triton X-100 (Sigma-Aldrich, St. Louis, MO, USA) for 10 min and then incubated in mouse anti-bovine cytokeratin-18-diluted antibodies (Abcam, Cambridge, United Kingdom) overnight at 4°C. The epithelial cells were washed three times in PBS and incubated with secondary antibody (goat anti-mouse IgG secondary antibody; Abcam) for 1 h at 37°C in the dark. After incubation, the cells were washed three times for 5 min with PBS in the dark. Subsequently, the nuclei were stained with 4′,6-diamidino-2-phenylindole (DAPI). The samples were observed on a Nikon Type 108 (Tokyo, Japan) laser confocal system.

### Preparation of the SQ0048 strain.

Frozen SQ0048 strain was subcultured in MRS broth under anaerobic incubation at 37°C for 12 h until the optical density at 600 nm (OD_600_) reached values approximating 1 ± 0.1, corresponding to 1.0 × 10^9^ CFU/ml. The SQ0048 strain was harvested by centrifugation at 4,000 rpm for 10 min at 4°C and washed with PBS solution. Thereafter, the SQ0048 strain was resuspended in antibiotic-free DMEM–F-12 medium (Gibco).

### Bacterial treatment.

BVECs were seeded at a density of 2 × 10^6^ cells/ml in 6-well cell culture plates and cultivated at 37°C and 5% CO_2_ until they reached confluence (day 1). Then, the cell culture supernatant was removed and 1 ml of fresh SQ0048 strain suspension at 1 × 10^9^ CFU/ml was added to the BVEC monolayer. After coculturing for 4 h at 37°C with 5% CO_2_, the supernatant was removed. The BVECs were gently washed three times with 1% sterile phosphate-buffered saline to release unbound bacteria and collected for transcription analysis. The control cells were treated identically with DMEM–F-12 medium. Three biological replicates were designed for the treatment and control experiments.

### RNA library construction and sequencing.

Total RNA from untreated and SQ0048 strain-treated BVECs was extracted using TRIzol reagent (Invitrogen/Thermo Fisher Scientific, Carlsbad, CA, USA) according to the manufacturer’s instructions. The quantity and purity of total RNA were analyzed using a Bioanalyzer 2100 and RNA 6000 Nano LabChip kit (Agilent, Santa Clara, CA, USA), yielding RNA integrity number (RIN) scores of >7.0. The cDNA libraries were constructed with RNA isolated from BVECs using a TruSeq stranded mRNA library preparation kit (Illumina, San Diego, CA, USA). To obtain mRNA, an approximately 5-μg sample of total RNA was used to isolate poly(A) mRNA with a poly(T) oligonucleotide primer attached to magnetic beads (Invitrogen). Sequencing libraries were generated using a New England BioLabs (NEB) Next Ultra RNA library prep kit for Illumina (NEB, Ipswich, MA, USA) according to the manufacturer’s instructions. To synthesize first-strand cDNA, purified mRNA was fragmented into small pieces, and then reverse transcriptase and random hexamer primers were utilized. Synthesis of second-strand cDNA was carried out using deoxynucleoside triphosphates containing DNA polymerase, dUTP, and RNase. End repair was achieved by adding dATP to all free 3′ ends, and unique index sequences for the fragments were added with adapter ligation. A sequencing library was obtained by PCR amplification of the products. The cDNA library was sequenced on an Illumina HiSeq 4000 sequencing platform (Illumina, San Diego, CA, USA) by LC Sciences (Hangzhou, China).

### Statistical analysis of transcription data.

The raw RNA-seq data were filtered to remove adapter contamination, low-quality bases, and undetermined bases utilizing Cutadapt software (47). Sequencing quality was verified using FastQC software, including the *Q*_20_, *Q*_30_, and GC content of the clean data. All downstream analysis was based on clean, high-quality data. The filtered reads were aligned and mapped to the reference genome using Hisat 2.0. The aligned read files were processed by Cufflinks (48), which uses the normalized RNA-seq fragment counts to measure the relative abundances of the transcripts. The unit of measurement is fragments per kilobases of exon per million fragments mapped (FPKM). The DEGs were screened using R package edger (49) with a fold change (FC) of ≥2 and a *P *value of <0.05 (50), which were considered significant differential expression.

The DEGs underwent enrichment analysis using GO and KEGG. *P* values were calculated using the Benjamini-corrected modified Fisher exact test, and *P < *0.05 was deemed statistically significant.

### Validation by quantitative reverse transcription-PCR.

RT-qPCR was utilized to validate the DEGs identified by RNA-seq. After treatment with DNase, the total RNA was reverse transcribed to produce cDNA using a reverse transcription system with a PrimeScript II 1st Strand cDNA synthesis kit (Vazyme, Nanjing, China). Fifteen DEGs were chosen based on changes in their expression levels in the treated cells compared with the control cells. The primer sequences, amplicon length, melting temperature, and GenBank accession numbers of both housekeeping and validated genes are summarized in Table 3. RT-qPCR amplification was performed on a Light Cycler 480 system (Roche, Basel, Switzerland) using the SYBR Green technique in 96-well optical plates. A PCR mix was prepared in a total volume of 25 μl: 8.5 μl water, 0.75 μl forward primer, 0.75 μl reverse primer, 2.5 μl cDNA, and 12.5 μl SYBR Green I master mix (Roche). The following amplification program was used: 5 min of preincubation at 95°C and 40 cycles of amplification for 10 s at 95°C for denaturation, 15 s at 58 to 60°C for annealing, and 20 s at 72°C for elongation. Negative controls (no cDNA) were run in the same reaction set. A dissociation stage was added to verify the presence of a gene-specific peak and the absence of primer-dimer peaks. The relative gene expression was determined using the threshold cycle (*C_T_*) method, and the fold changes were calculated using the 2^−ΔΔ^*^CT^* formula (51).

**TABLE 3:** Primers for RT-qPCR

Gene name	Nucleotide sequence (5′ to 3′)
β-Actin	Forward TCACCAACTGGGACGACA
	Reverse GCATACAGGGACAGCACA
CKAP4	Forward CCTGCTGGACAGACTCTCCTCTC
	Reverse GGCGGACTCCAAGTTGTTCTCG
DNAJB1	Forward CACCGAAGAACTCAGCAAAC
	Reverse ACCACCCGGACAAGAACA
DNAJA1	Forward TTCTACCTGGTCCATTTC
	Reverse CAACCGAACCATAGTCAT
HSPH1	Forward AACTCAACATTCACCACC
	Reverse TATCCAGCAAGACAACAA
HSPA8	Forward GGATGACACCTCCTCTGG
	Reverse CGCCTTTACTGATACCGA
PPP1R15A	Forward GAGGTGGGAGCCTACAGA
	Reverse GGTGGAGGTAACGAAGTGA
FOSL1	Forward CACAGGCCAGCAGAAGTTCCAC
	Reverse TGAGGTTGAACCATCCACTGAAGC
JUN	Forward TGCCCGTTGCTGGACTGTAT
	Reverse ACGACCTTCTACGACGATGCC
IL17RA	Forward TTCTGGATTGGTGGTTTGG
	Reverse TGCCTTCAGCCACTTCGT
FOSB	Forward CCCAGCCTTGACCGTAGAT
	Reverse CGAGAAGTTTGCCGAGTGA
CD74	Forward CAGCCCAGATGACGGATA
	Reverse AGCCCAACTGCGACGAGA

### Statistical analysis.

The experimental data were expressed as mean ± SD. The difference between two groups was analyzed using the two-tailed Student *t* test. *P < *0.05 was considered significant with * indicating *P < *0.05 and ** indicating *P < *0.01.

### Data availability.

The sequencing data determined in this work have been deposited in the NCBI GEO public database (https://www.ncbi.nlm.nih.gov/gds/?term=) and are accessible through GEO series accession number GSE127808.
